# Human Gut Microbiota Associated with Obesity in Chinese Children and Adolescents

**DOI:** 10.1155/2017/7585989

**Published:** 2017-10-29

**Authors:** Ya-Ping Hou, Qing-Qing He, Hai-Mei Ouyang, Hai-Shan Peng, Qun Wang, Jie Li, Xiao-Fei Lv, Yi-Nan Zheng, Shao-Chuan Li, Hai-Liang Liu, Ai-Hua Yin

**Affiliations:** ^1^Medical Genetic Centre, Guangdong Women and Children Hospital, Guangzhou, Guangdong 510010, China; ^2^Maternal and Children Metabolic-Genetic Key Laboratory, Guangdong Women and Children Hospital, Guangzhou, Guangdong 510010, China; ^3^CapitalBio Genomics Co., Ltd., Dongguan 532808, China; ^4^Department of Pediatrics, Guangdong Women and Children Hospital, Guangzhou, Guangdong 510010, China

## Abstract

**Objective:**

To investigate the gut microbiota differences of obese children compared with the control healthy cohort to result in further understanding of the mechanism of obesity development.

**Methods:**

We evaluated the 16S rRNA gene, the enterotypes, and quantity of the gut microbiota among obese children and the control cohort and learned the differences of the gut microbiota during the process of weight reduction in obese children.

**Results:**

In the present study, we learned that the gut microbiota composition was significantly different between obese children and the healthy cohort. Next we found that functional changes, including the phosphotransferase system, ATP-binding cassette transporters, flagellar assembly, and bacterial chemotaxis were overrepresented, while glycan biosynthesis and metabolism were underrepresented in case samples. Moreover, we learned that the amount of* Bifidobacterium* and* Lactobacillus* increased among the obese children during the process of weight reduction.

**Conclusion:**

Our results might enrich the research between gut microbiota and obesity and further provide a clinical basis for therapy for obesity. We recommend that* Bifidobacterium* and* Lactobacillus* might be used as indicators of healthy conditions among obese children, as well as a kind of prebiotic and probiotic supplement in the diet to be an auxiliary treatment for obesity.

## 1. Introduction

Obesity is a condition mainly caused by an alteration in energy intake, shifting towards positive energy balance, which can be influenced by genetic and environmental factors [1]. The prevalence of obesity is increasing at an epidemic rate globally, with more than one billion adults overweight and at least 300 million of them clinically obese [2]. Morbid obesity is known to be accompanied by serious health conditions, including hypertension, type 2 diabetes, cardiovascular diseases, stroke, and venous thromboembolism [3].

A large number of diverse microbial species reside in the distal gastrointestinal tract, and gut microbiota dysbiosis results in the imbalance of the composition and function of these intestinal microbes [4]. To date, many studies demonstrate that gut microbes play a great role in disease, ranging from localized gastroenterologic disorders to neurologic, respiratory, hepatic, and cardiovascular illness and metabolic related syndromes [5–8]. In particular, the fundamental role of gut microbiota in regulation and pathogenesis of metabolic disorders has attracted interest of more and more researchers in recent years. For the aspect of obesity, the research team of Jeffrey Gordon learned that differences of gut microbial communities were associated with obesity and that obesity would lead to reducing the diversity of microorganisms [9]. Subsequently, they confirmed the great role of gut microbes in obesity with an animal model in mice [10]. Dietary habits have strong influences on the selection of gut microbiota. Recently, proposed mechanisms about how the gut microbiota contributes to obesity, including the microbiota of obese subjects, had higher capacity to harvest energy from the diet providing substrates that can activate lipogenic pathways and, in addition, influence the activity of lipoprotein lipase interfering in the accumulation of triglycerides in the adipose tissue [11]. The gut microbiota has been proposed as another environmental factor involved in the onset of obesity. However, to which extent and through which mechanism the gut microbiota contributes to obesity development have not yet been elucidated.

In this present study, we recruited 87 obese children and adolescents as the case group and 56 healthy children and adolescents as the control group to analyze the community composition of gut microbiota between them and to further reveal potential microbial functional characteristics of obese children.

## 2. Method and Materials

### 2.1. Cohort in This Study

A total of 87 obese children (3–18 years) were enrolled in this study. In addition, 56 healthy children (3–18 years) were recruited as the healthy controls. Eleven of the obese children received an intervention in the hospital according to a dietary formula used in a previous study [12] and were resampled 30, 60, and 90 days after the intervention. None of the cohorts took antibiotics within the 3 months prior to sampling.

### 2.2. DNA Extraction and High-Throughput Sequencing

Community DNA was extracted from fecal samples using TIANamp Stool DNA Kit (TIANGEN, China), according to the manufacturer's instructions. We amplified the V3 region of the 16S rRNA gene by PCR with the following cycling conditions: a denaturing cycle of 98°C for 2 min, followed by 35 cycles of 94°C for 30 s, 55°C for 30 s, and 72°C for 30 s. Amplified products were recycled with Agencourt AMPure XP kit and the PCR products were sequenced with the Ion PGM platform (Life, USA).

### 2.3. Data Processing

We filtered out the reads below the criteria of read quality not less than 25, length between 100 and 500 bp, homopolymers less than 6, and mismatches no more than 2. High-quality reads were clustered to operational taxonomic units (OTUs) (closed-reference) and annotated with taxonomy information from Greengene (ver.13_5) using QIIME pipeline [13]. Reads assigned to OTUs were rarefied with 20,000 per library (minimum reads among all samples) and then normalized by the 16S rRNA gene copy numbers using PICRUSt [14]. We generated a KO (Kyoto Encyclopedia of Genes and Genomes (KEGG) orthology) profile with PICRUSt and grouped KOs into metabolic pathway levels for subsequent statistical analysis.

### 2.4. Statistical Analysis

Principal coordinate analysis (PCoA) was performed with weighted-UniFrac distance matrix of all normalized OTUs through QIIME. The OTUs with occurring frequency among all time-scale samples over 40% were set as core OTUs. We clustered the core OTUs using the Ward clustering algorithm based on Spearman correlation coefficient distance matrix (1 − correlation coefficient) and determined the* P* values for each node with the Permutational MANOVA method (9999 permutations,* P* < 0.01) using R package vegan. Genus-level markers were identified with Wilcoxon's rank sum test and effect sizes of LDA algorithm using LEfSe [15].

## 3. Results

In this study, the 16S rRNA gene V3 hypervariable region was sequenced on the Ion PGM platform. We generated 8,580,178 raw reads with a median read length of 175 base pairs. After quality trimming and chimer filtering, 7,061,487 high-quality sequences remained and accounted for an average of 40,122 reads (range from 20,103 to 62,253) per sample. When we randomly selected 20,000 reads 10 times from all samples, the median average value of Good's coverage was nearly 98.65%, which was close to the plateau (see Figure S1 in Supplementary Material available online at https://doi.org/10.1155/2017/7585989), indicating the sequencing depth was sufficient for characterizing the bacterial population in our fecal samples.

### 3.1. Gut Microbiota Dysbiosis in Obese Children

**Figure 1:**
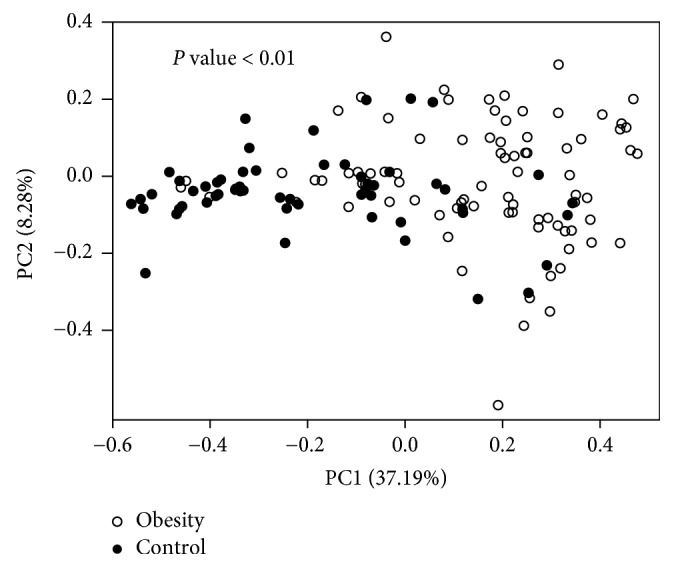
*Composition comparison of fecal microbiota between the obese and control group*. Plot of fecal microbiota based on the weighted UniFrac metric with principal coordinate analysis results.

To determine how the gut microbial community shifts in obese children, we compared the overall structure of the fecal microbiota in the obese population with that of the healthy control population. Unexpectedly, obese children, not obese adults, did not exhibit a significant decrease in the fecal community diversity and richness, but the community structure of the fecal microbiota was quite different between the obese and healthy control group. Weighted-UniFrac distance was calculated to measure the similarity between microbial communities, which considered both the composition structure and the abundance of microbiota. Noticeably, the fecal microbiota from the obese group could be separated from those of the control group with principal coordinate analysis (*P* value < 0.01, Figure 1).

### 3.2. Obese-Associated Gut Microbes in Children

The taxonomy of the fecal microbiota was assessed by a taxon-dependent analysis. Eighteen phyla were detected in our fecal samples, including 13 phyla observed in the obese cohort and 16 phyla observed in the normal control cohort (Figure S2). Firmicutes and Bacteroidetes were the predominant fecal microbiome in both cohorts. However, the relative abundance ratio of Firmicutes and Bacteroidetes (F/B) in the obese cohort was significantly higher than in the normal control cohort. The phyla of Verrucomicrobia, TM7, and Lentisphaerae had a median relative abundance < 0.1%, which was significantly lower than those in the control group.

**Figure 2:**
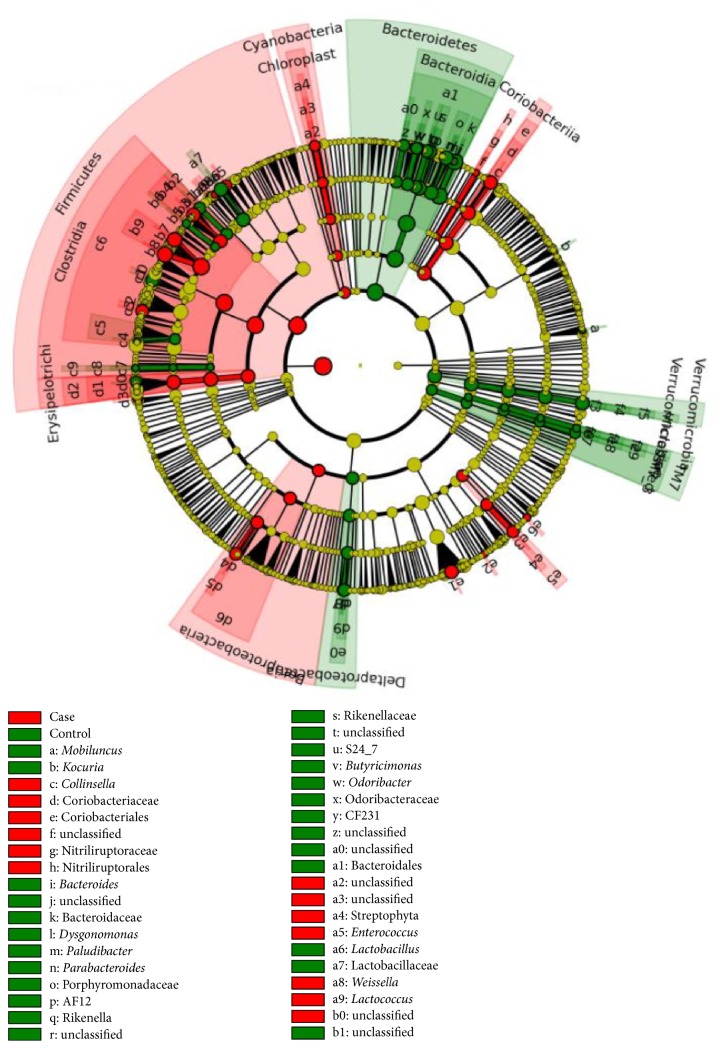
*Community population differences between the obese and control group*. Enrichment microbes from obese samples are marked red, while the control samples are marked green.

To identify the specific bacteria taxa associated with childhood obesity, we compared OTUs in case and control groups using LEfSe. A cladogram representative of the structure of the fecal microbiota was shown in Figure 2. The data indicated that intestinal dysbiosis was extensive in obese children. In the obese children, microbial assemblages were notably enriched in Firmicutes, especially* Enterococcus* and* Blautia*, as well as* Sutterella* and* Klebsiella* from Proteobacteria and* Collinsella* from Actinobacteria, while* Bacteroides* and* Parabacteroides* from Bacteroidetes, as well as* Anaerotruncus* and* Coprobacillus* from Firmicutes were enriched in healthy controls (data in Table S1).

### 3.3. Potential Microbial Functional Characteristics of Obese Children

**Figure 3:**
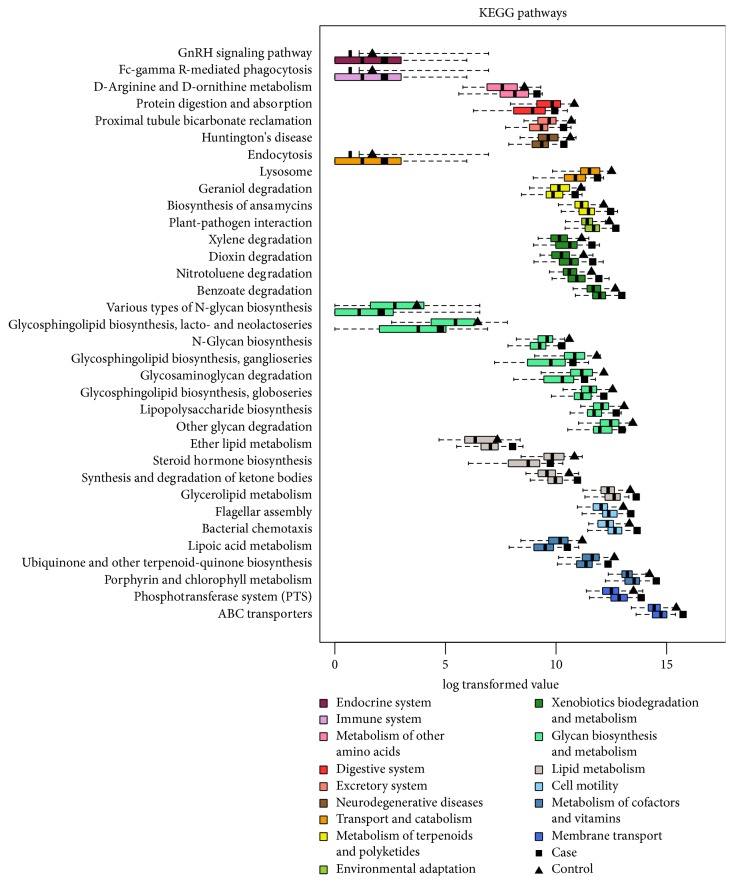
*Functional changes derived from the obesity-associated gut microbiota*. All significantly different level 3 pathways are illustrated and colored according to level 2 pathway categories. Ordinate indicated metabolic pathways; different colors represent the classification of different metabolic pathways. Each pathway has two box figures, which represent the expression of obese children and control samples in this metabolic pathway. Significant changes in the relative abundance of estimated genes summarized by KEGG pathway annotations between obese children and controls are shown with* P* values < 0.05.

To investigate the relationship between obese and gut microbiome functions, we predicted the potential metagenomes from the community profiles of normalized 16S rRNA genes with PICRUSt [14]. The inferred gene families (KEGG Orthology groups) were annotated and combined with level 2 and level 3 pathways for statistic testing and visualization (Figure 3 and Table S2). Combined KEGG pathways and PICRUSt analysis results included categories associated with membrane transport and cell motility, which were overrepresented, while glycan biosynthesis and metabolism were underrepresented in case samples. Level 3 pathways in membrane transport consisted of the phosphotransferase system (PTS), ATP-binding cassette transporters (ABC transporters), and cell motility that contained flagellar assembly and bacterial chemotaxis.

### 3.4. Assessing the Functional Gut Microbes after Taking Dietary Therapy

**Figure 4:**
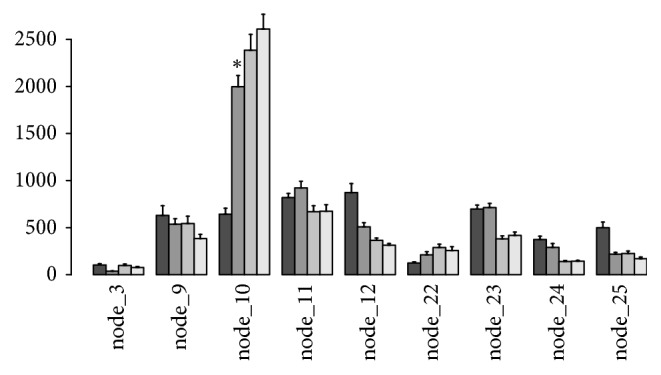
*Abundance results of significant clusters of core OTUs for samples during 0, 30, 60, and 90 days of the dietary intervention*. The asterisk indicates the statistical significance of* P* value < 0.01. OTUs species information in node_10 is listed in Table S3. ^*∗*^indicates the statistical significance of *P* value <0.01 compared with the dark grey sample (as control) in node_10.

Eleven of the obese children in our study received an intervention in the hospital according to a dietary formula used in a previous study and were resampled 30, 60, and 90 days after the intervention. When analyzed, we divided the core OTUs into groups by Ward's hierarchical clustering based on Spearman's correlation distance. Each node of the dendrogram was tested using a permutational method and nine significant nodes (clusters) were retrieved. We found that the abundance of Node 10 displayed significant differences among samples of 0, 30, 60, and 90 days during the process of weight reduction (Wilcoxon's rank sum test,* P* < 0.01) (Figure 4). Node 10 mostly comprised members of the genus* Bifidobacterium* and* Lactobacillus* (Table S3).

## 4. Discussion

Obesity is a metabolic disease that is often accompanied by dyslipidemia, hypertension, and impaired glucose homeostasis, and it has been reported to be associated with intestinal dysbiosis [1, 16]. In knockout and diet-induced obese mice, obesity was associated with changes in the composition and metabolic function of the gut microbiota [17]. Dysbiosis is a form of altered gut metagenome and collected microbial activities, and, in combination with classic genetic and environmental factors, it may promote the development of metabolic disorders [18]. To deeply understand obesity and the metabolic mechanism related to gut microbiota would make an improvement in the treatment of obesity.

### 4.1. Gut Microbiota Dysbiosis in Obese Children

Here, we recruited 87 obese children and adolescents as a case group and 56 healthy children and adolescents as a control group to analyze the gut microbiota differences between them. Based on our results, obese children here did not exhibit a significant decrease in the fecal community diversity and richness, while being different from the results based on obese adults. However, the composition of the gut microbiota showed significant differences between obese children and healthy controls. First, our results indicated that the phyla of Firmicutes and Bacteroidetes were the predominant fecal microbiome in both cohorts. However, the relative abundance ratio of Firmicutes and Bacteroidetes (F/B) in the obese cohort was significantly higher than that in the healthy controls (Figure S2), which was consistent with the study in obese adults [19]. Firmicutes is associated with genes involved in carbohydrate catabolism and is rich in obese individuals [20], while Bacteroidetes is linked with diminished body mass [21]. Second, we also found some specific bacteria exhibited significant intergroup variations in their abundance, with changes in* Enterococcus*,* Blautia*,* Sutterella*,* Klebsiella,* and* Collinsella*, which were enriched in obese children, while* Bacteroides*,* Parabacteroides*,* Anaerotruncus,* and* Coprobacillus* were enriched in healthy controls (Table S1). Turnbaugh et al. also found that after mice were fed a low-fat, plant polysaccharide-rich diet and then switched to a Western diet, the microbiota composition shifted to an overgrowth of Firmicutes, including* Clostridium innocuum*,* Eubacterium dolichum*,* Catenibacterium mitsuokai,* and* Enterococcus* spp., and to a significant reduction in several* Bacteroides* spp. [22]. Case-specific markers in our study were found to be associated with a high-fat Western diet from previous research and might be a contributing factor to obesity. In addition, bacteria belonging to the genera* Collinsella* have been reported to be positively correlated with insulin [23] and that it was enriched in T2D patients [24]; therefore, enrichment of* Collinsella* here might imply the possibility of insulin resistance in obese children. In addition,* Blautia producta* was suggested to be related to a high-fat diet causing obesity in a mouse model [25].

### 4.2. Obese Related Functional Change

Intestinal dysbiosis in obese children would lead to functional change. With the KEGG pathway results (Figure 3 and Table S2), we knew that the activities of membrane transport and cell motility were overrepresented, while that of glycan biosynthesis and metabolism were underrepresented in case samples. Among these signaling pathways, phosphotransferase systems from the related pathway of membrane transport were more active in obese children. Phosphotransferase systems (PTS) are a class of transport systems involved in the uptake and phosphorylation of a variety of carbohydrates. The PTS also plays a role in regulating microbial gene expression through catabolite repression, allowing the cell to preferentially import simple sugars over other carbohydrates [26, 27]. Moreover, functional analysis revealed that the obese human gut microbiome was enriched with phosphotransferase systems involved in microbial processing of carbohydrates primarily present in Actinobacteria and Firmicutes [9, 28]. In addition, ATP-binding cassette transporters (ABC transporters) from the related pathway of membrane transport were also active. ABC transporters are members of a transport system super family in all extant phyla from prokaryotes to humans [29]. ABC uptake transporters could uptake a large variety of nutrients, biosynthetic precursors, trace metals, and vitamins, while exporters transport sterols, lipids, drugs, and a large variety of primary and secondary metabolites. Some of these exporters in humans are involved in tumor resistance, cystic fibrosis, and a range of other inherited human diseases [30]. The ABC transporter related diseases include Alzheimer's disease and drug resistance of malignant tumors [31, 32]. However, this was the first time to be mentioned that ABC transporters have a relationship with obesity, here. More evidence and further mechanisms need to be provided next.

Second, cell motility, which involved flagellar assembly, and bacterial chemotaxis were second active in obese children. In 2009, Hildebrandt et al. found that a collection of gene groups linked to bacterial motility increased on a high-fat diet, including “flagellar motility” and “motility and chemotaxis” [33].

Last, biosynthesis of various types of N-glycans, glycosphingolipids, lipopolysaccharide (LPS), and degradation of glycosaminoglycans (GAGs) and other glycans were found to be lower here in obese children compared with the healthy controls. N-glycan biosynthesis disorders could cause metabolic diseases, such as classical galactosemia (OMIM #230400) [34]. Glycan biosynthesis and metabolism disorders could lead to a wide range of carbohydrate imbalance in the human body, hence, being known to play a great role in change in weight reduction [35]. LPS is a cell wall component of Gram-negative bacteria, which lyses into plasma when the bacteria die. Here, the phyla of Verrucomicrobia and Lentisphaerae, belonging to Gram-negative bacteria, were significantly lower in obese group than those in the control group, which may lead to lower level of LPS in the obese children. Cani et al. found that LPS, which acts on the innate immune system response in adipose tissue in obesity, is the triggering factor of the early development of inflammation and metabolic diseases [36]. Importantly, Verrucomicrobia acts as a polysaccharide degrader and is proposed as a hallmark of a healthy gut due to its anti-inflammatory and immunostimulant properties and its ability to improve gut barrier function, insulin sensitivity, and endotoxinemia [37]. Researchers studied that the inflammation is not all bad for obesity, and it would play a positive role by stimulating energy expenditure and facilitating adipose tissue remodeling [38, 39]. Obesity is associated with low-grade inflammation in adipose tissue, which has been implicated in the development of metabolic syndrome and insulin resistance. Therefore, low-LPS could disrupt the energy consumption and adipose tissue remodeling.

### 4.3. Gut Microbiota Changes under the Process of Weight Reduction

Upon analyzing the gut microbiota changes after 0, 30, 60, and 90 days during the process of weight reduction in eleven obese children, who in our study received an intervention in a hospital according to a dietary formula used in a previous study, we found that the abundance of Node 10 displayed significant differences (*P* value < 0.01) (Figure 4), which mostly contained members of the genus* Bifidobacterium* and* Lactobacillus* (Table S3). As reported before, dietary oligosaccharides derived from plant and milk stimulate the growth of* Bifidobacterium*, which is linked to normal weight gain and were found in low concentrations in obese individuals [40].* Bifidobacterium* could reduce obesity-associated inflammation by restoring the lymphocyte-macrophage balance and reduce the abundance of Firmicutes, in which these effects were accompanied by reductions in body weight gain and in serum cholesterol, triglycerides, glucose, and insulin levels and improved oral glucose tolerance and insulin sensitivity in obese mice [41]. In addition,* Lactobacillus* played a dual function in preventing high-fat-diet-induced obesity, including direct reduction of cholesterol and upregulation of PPARalpha in adipose tissue [42, 43]. Therefore, promoting changes in the gut microbiota might play an important role in ensuring the efficacy and success of obesity treatments. To our knowledge,* Bifidobacterium* and* Lactobacillus* might be used as indicators of healthy conditions among obese children and adolescents, as well as a kind of prebiotic and probiotic supplement in the diet to be an auxiliary treatment in obesity.

## 5. Conclusion

In summary, from the present study, we learned that the gut microbiota composition was significantly different between obese children and the healthy cohort, which included bacteria categories of* Enterococcus*,* Blautia*,* Sutterella*,* Klebsiella,* and* Collinsella*, which were enriched in obese children, while* Bacteroides*,* Parabacteroides*,* Anaerotruncus,* and* Coprobacillus* were enriched in healthy controls. Then, we knew that functional changes, including PTS and ABC transporter related membrane transport and flagellar assembly and bacterial chemotaxis related cell motility, were overrepresented, while glycan biosynthesis and metabolism were underrepresented in case samples. Moreover, we learned that the amount of* Bifidobacterium* and* Lactobacillus* increased among the obese children during the process of weight reduction. Our results might enrich the research between gut microbiota and obesity, further providing a clinical basis for therapy for obesity. Finally, we recommend that* Bifidobacterium* and* Lactobacillus* might be used as indicators of healthy conditions among obese children and adolescents, as well as a kind of prebiotic and probiotic supplement in the diet to be an auxiliary treatment for obesity.

## Supplementary Material

Fig. S1: Rarefaction curve of Good's coverage estimator with random resampling.
Fig. S2: The difference in relative abundance of the gut microbiota between obese children and the healthy cohort at the phyla level.
Table S1: Lefse-selected biomarkers at the genus level.
Table S2: P values of Wilcoxon's rank sum test results for KEGG pathways at the level 3.
Table S3: Taxonomy annotations of OTUs within the node 10.
