# Supplementary figures and images for: Human Gut Microbiota Associated with Obesity in Chinese Children and Adolescents
Source: Biomed Res Int. 2017 Oct 29;2017:7585989. doi: 10.1155/2017/7585989 (PMC5682041; doi:10.1155/2017/7585989)

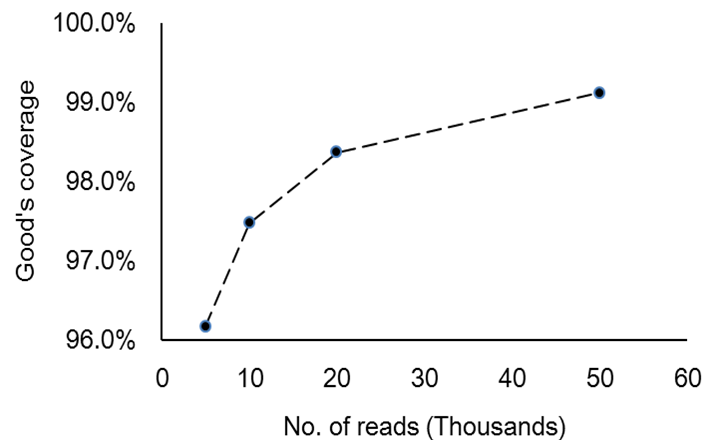

Supplement: Supplementary file 1 — Fig. S1: Rarefaction curve of Good's coverage estimator with random resampling. Fig. S2: The difference in relative abundance of the gut microbiota between obese children and the healthy cohort at the phyla level. Table S1: Lefse-selected biomarkers at the genus level. Table S2: P values of Wilcoxon's rank sum test results for KEGG pathways at the level 3. Table S3: Taxonomy annotations of OTUs within the node 10. [file 7585989.f1.zip › Fig S1.tif]

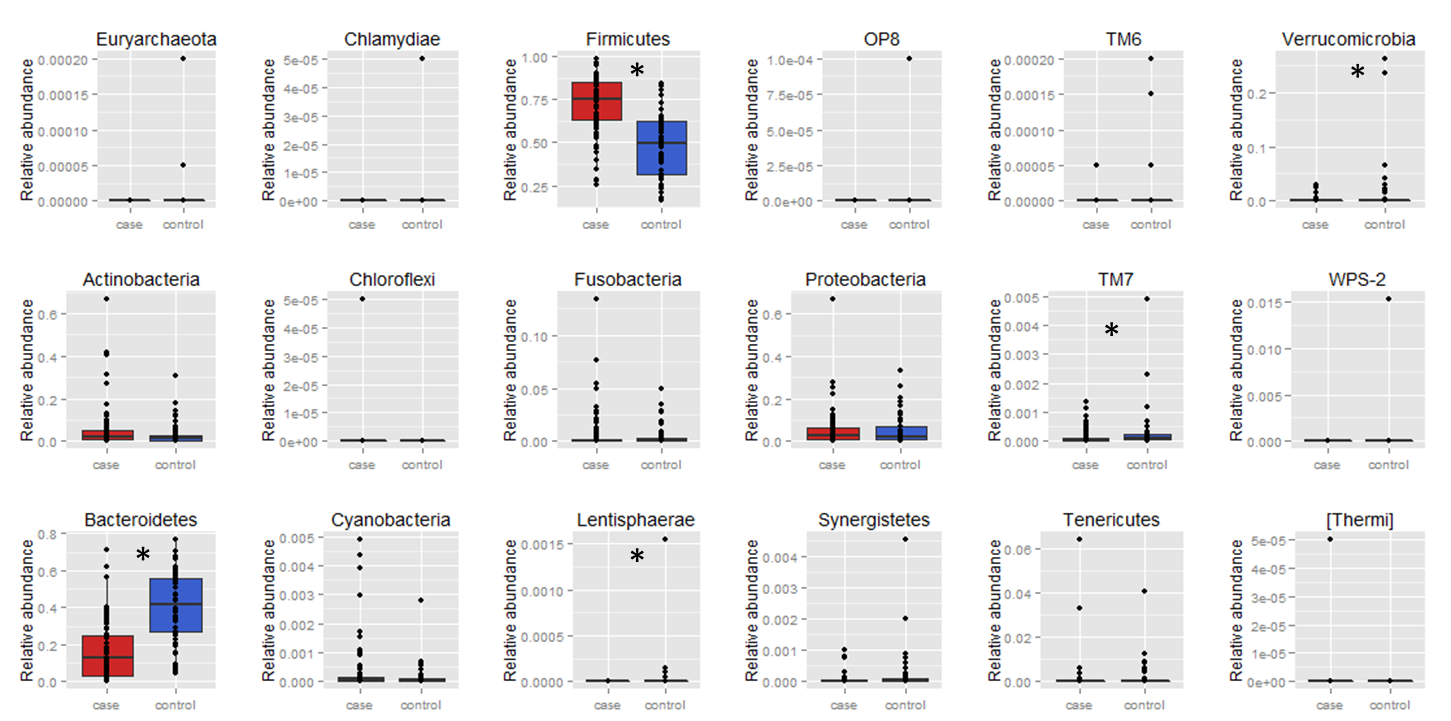

Supplement: Supplementary file 1 — Fig. S1: Rarefaction curve of Good's coverage estimator with random resampling. Fig. S2: The difference in relative abundance of the gut microbiota between obese children and the healthy cohort at the phyla level. Table S1: Lefse-selected biomarkers at the genus level. Table S2: P values of Wilcoxon's rank sum test results for KEGG pathways at the level 3. Table S3: Taxonomy annotations of OTUs within the node 10. [file 7585989.f1.zip › Fig S2.tif]
